# K_V_1.5–K_V_β1.3 Recycling Is PKC-Dependent

**DOI:** 10.3390/ijms22031336

**Published:** 2021-01-29

**Authors:** Alvaro Macias, Alicia de la Cruz, Diego A. Peraza, Angela de Benito-Bueno, Teresa Gonzalez, Carmen Valenzuela

**Affiliations:** 1 Instituto de Investigaciones Biomédicas Madrid CSIC-UAM. C/Arturo Duperier 4, 28029 Madrid, Spain; axd1352@med.miami.edu (A.d.l.C.); diego.dayago@gmail.com (D.A.P.); adebenito@iib.uam.es (A.d.B.-B.); tgonzalez@iib.uam.es (T.G.); 2 CIBER Enfermedades Cardiovasculares (CIBERCV), Madrid, Spain; 3 Department of Biochemistry, School of Medicine, Universidad Autónoma de Madrid, 28029 Madrid, Spain; 4 Instituto de Investigación Sanitaria del Hospital Universitario La Paz (IdiPAZ), 28029 Madrid, Spain

**Keywords:** K_V_1.5, K_V_β1.3, PKC, calphostin C, RACK1, bisindolylmaleimide II, traffic

## Abstract

K_V_1.5 channel function is modified by different regulatory subunits. K_V_β1.3 subunits assemble with K_V_1.5 channels and induce a fast and incomplete inactivation. Inhibition of PKC abolishes the K_V_β1.3-induced fast inactivation, decreases the amplitude of the current K_V_1.5–K_V_β1.3 and modifies their pharmacology likely due to changes in the traffic of K_V_1.5–K_V_β1.3 channels in a PKC-dependent manner. In order to analyze this hypothesis, HEK293 cells were transfected with K_V_1.5–K_V_β1.3 channels, and currents were recorded by whole-cell configuration of the patch-clamp technique. The presence of K_V_1.5 in the membrane was analyzed by biotinylation techniques, live cell imaging and confocal microscopy approaches. PKC inhibition resulted in a decrease of 33 ± 7% of channels in the cell surface due to reduced recycling to the plasma membrane, as was confirmed by confocal microscopy. Live cell imaging indicated that PKC inhibition almost abolished the recycling of the K_V_1.5–K_V_β1.3 channels, generating an accumulation of channels into the cytoplasm. All these results suggest that the trafficking regulation of K_V_1.5–K_V_β1.3 channels is dependent on phosphorylation by PKC and, therefore, they could represent a clinically relevant issue, mainly in those diseases that exhibit modifications in PKC activity.

## 1. Introduction

In humans the ultrarapid outward potassium current (*I_Kur_*) is only present in atria and it is generated after the activation of K_V_1.5 channels together with other regulatory subunits (K_V_β1.2, K_V_β1.3 and K_V_β2.1). Its kinetics and voltage dependence underlie their key role in the regulation of the atrial action potential duration [1,2]. The *I_Kur_* slow inactivation may be modified by the assembly of K_V_1.5 channels with β subunits present in the human myocardium [3,4]. The K_V_β1.3 subunit induces fast and partial inactivation, greater degree of slow inactivation, slower deactivation process and negative shift of the activation curve [5,6,7]. Moreover, K_V_β1.3 decreases by 7-fold the sensitivity of these channels to block inducement by antiarrhythmic drugs and local anesthetics and almost abolish the stereoselectivity of bupivacaine block [6,8,9].

We have previously demonstrated that K_V_1.5–K_V_β1.3 channels interact with RACK1, PKCβI, PKCβII, PKCθ, PLC and PIP_2_, either directly or through scaffold proteins, generating a K_V_1.5 *channelosome*, observed both in HEK293 cells and in rat ventricular tissue [7], and also that the inhibition of PKC reverted the pharmacological properties of K_V_1.5–K_V_β1.3 channels towards K_V_1.5 channels [10]. The knowledge of the proteins forming such *channelosome* may explain the differences between *I_Kur_* recorded in ventricle and atrium in different animal species and open up the discovery of new drug targets useful for the treatment of several cardiomyopathies. Upon activation, most PKC isoforms undergo specific compartmentation that afterwards is fine-tuned by interactions with specific PKC adaptor proteins (RACKs) [11]. The function of these adaptor proteins is to translocate bound proteins to distinct subcellular locations through their protein–protein interaction domains (WD40) [12], allowing them to regulate different cellular responses [13].

In the present study, we have studied the effects of PKC inhibition on the recycling of Kv1.5–Kvβ1.3 complexes. Our experiments show that PKC activity is necessary not only for the biophysics and pharmacological activity of K_V_1.5–K_V_β1.3 complex, but also for their recycling. We observed that PKC inhibition both decreases the amount of K_V_1.5–K_V_β1.3 channels in the membrane and increases their levels in the cytoplasm, suggesting that PKC activity is necessary for a correct recycling of these channels.

## 2. Results

### 2.1. PKC Inhibition Reduces the Surface Amount of K_V_1.5 Channel

**Figure 1:**
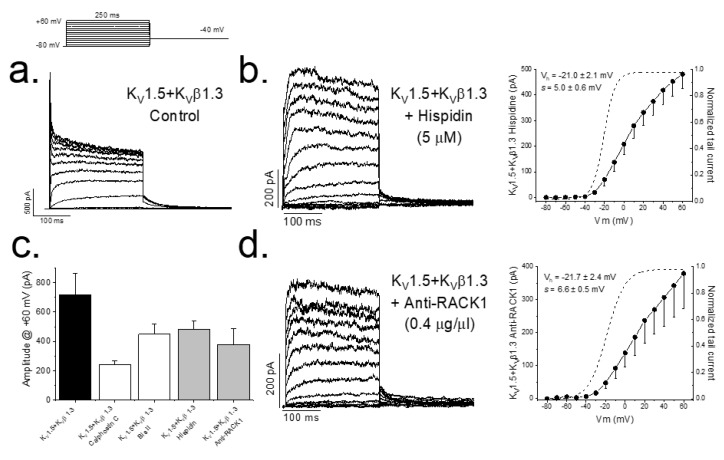
PKC inhibition and RACK1 block results in a smaller magnitude of the K_V_1.5–K_V_β1.3 current. Representative currents and I–V relationships in control conditions (**a**) along the activation curves, in a dashed line (right), after PKC inhibition with hispidin (**b**) and in the presence of anti-RACK1 antibody into the internal solution (**d**). Graphs shown in (**c**) show a summary of the magnitude K_V_1.5–K_V_β1.3 current measured at the end of +60 mV depolarizing pulses under control conditions and after inhibition of PKC (with different inhibitors) and after RACK1 block with anti-RACK1 present in the internal solution. Note that data for calphostin C and bisindolylmaleimide II are taken from data previously reported in Macias et al., 2014 [10].

In previous studies, we have reported that either inhibition of PKC (by calphostin C or bisindolylmaleimide II) or silencing the expression of PKC is able to abolish the K_V_β1.3-induced fast inactivation as well as modify the pharmacology of the channel [7,10]. Figure 1 also shows that PKC inhibition produces a smaller magnitude of the current (from 719.2 ± 145.2 pA (*n* = 14) to 240.4 ± 26.5 pA (*n* = 41) in control conditions and after treatment with calphostin C for 2 h, respectively, *p* < 0.05). The inhibition of PKC activity with hispidin (Figure 1a) or after preventing the arrangement of the PKC–RACK1 complex, essential for PKC activity, by adding anti-RACK1 to the patch pipette (Figure 1b) also eliminated the K_V_β1.3-induced fast inactivation. In these experiments, the magnitude of the current was also smaller than under control conditions, similar to that observed in those experiments in which PKC was inhibited with calphostin C, without changes in the activation curves (Figure 1c).

**Figure 2:**
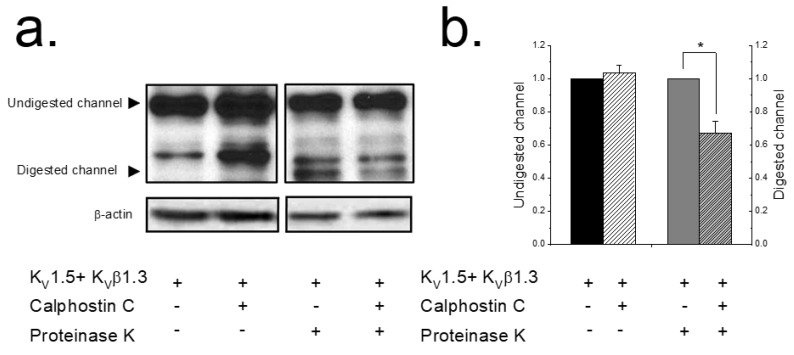
PKC inhibition decreases the K_V_1.5–K_V_β1.3 levels in the plasma membrane. HEK293 **c**ells expressing K_V_1.5–K_V_β1.3 channels treated or not with calphostin C (3 μM for 2 h). Cells were treated with buffer (lanes 1 and 2) or with proteinase K externally (lanes 3 and 4). Left panel shows a representative experiment (**a**). The two arrows indicate the molecular weight at which digested K_V_1.5 (surface channel) and undigested K_V_1.5 (intracellular form) appear. β-actin was used as a loading control. Densitometric analysis of 5 experiments as those shown in panel **a**. Black bars represent, normalized to control, total protein levels; grey bars, normalized to control, proteinase-K-digested K_V_1.5–K_V_β1.3 channel as a representation of plasma membrane levels (**b**). Each value represents the mean ± SEM of five experiments. * *p* < 0.05.

These results could be explained by a lesser amount of K_V_1.5 channels at the plasma membrane after inhibiting PKC. To assess this hypothesis, an extracellular enzymatic digestion of the K_V_1.5–K_V_β1.3 channels using proteinase K followed by a Western blot analysis was performed (Figure 2) as previously described [14,15]. When applying externally proteinase K to living cells, this enzyme cleaves the protein on the external part of the cell, whereas the cytoplasmic proteins remain unaffected. This approach is a very sensitive test for analyzing plasma membrane protein expression since, as is shown in Figure 2a, cytoplasmic proteins are unaffected during the enzymatic treatment. Uncleaved Kv1.5 migrates at 75 kDa and digested channels migrate at ~45 kDa. A densitometric analysis of the results shown in Lanes 3 and 4 of Figure 2b demonstrated that, compared with non-treated cells, the intensity of the 45 kDa Kv1.5 band decreased in cells expressing K_V_1.5–K_V_β1.3 treated with calphostin C. Figure 2b shows the quantification of the digested channel demonstrating a significant decrease in the amount of channel in the plasma membrane after 2 h of treatment with calphostin C (32.6 ± 7.1%, *n* = 5, *p* < 0.05).

### 2.2. PKC Inhibition Increases the Internalized K_V_1.5–K_V_β1.3 Channels

**Figure 3:**
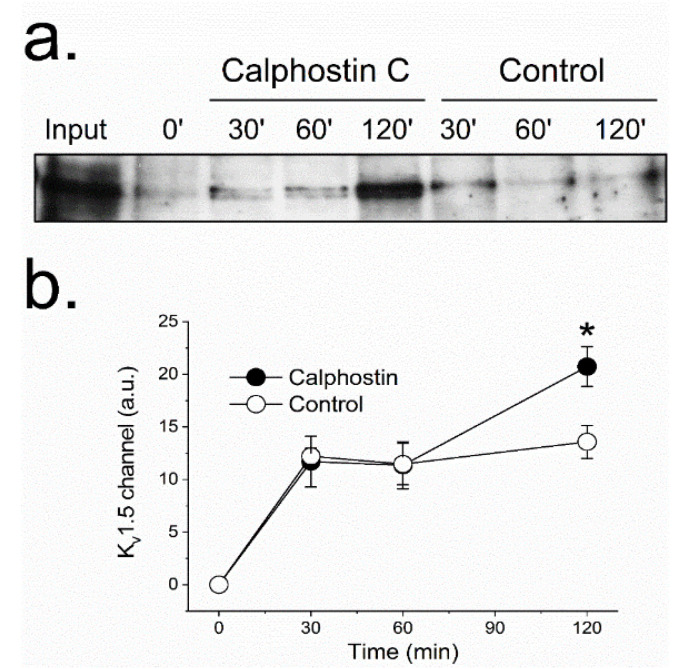
PKC inhibition modifies the K_V_1.5 recycling. Recycling was analyzed with a biotinylation test (see Materials and Methods). Western blot of internalized channels (biotinylated) at different times (30, 60 and 120 min) under control and after PKC inhibition with calphostin C (**a**). Densitometric analysis of all the experiments performed (**b**). Each value represents the mean ± SEM of three experiments. * *p* < 0.05.

The reduction observed in the K_V_1.5–K_V_β1.3 protein at the cell membrane after PKC inhibition suggests changes in the K_V_1.5–K_V_β1.3 trafficking. These modifications may be explained either by (1) an increase in the K_V_1.5–K_V_β1.3 channels endocytosis, or (2) a reduction of the endosomes-to-plasma membrane recycling of these channels. To be able to discriminate between these two possible explanations, we performed a series of biotinylation experiments (Figure 3). In these experiments, to increase the biotin binding to the channel, we used a K_V_β1.3–K_V_1.5–FLAG construct, which exhibited similar electrophysiological characteristics to channels without the extracellular FLAG motif [16]. The results provided by these assays suggest that during the first 60 min, the internalization dynamics of K_V_1.5–K_V_β1.3 channels in cells whose PKC was inhibited (with calphostin C) was similar to that observed under control conditions. Nevertheless, cells in which PKC was inhibited showed a significant increase in the amount of intracellular channels after two hours of treatment (13.6 ± 1.6% vs. 20.7 ± 1.9% in control and calphostin-C-treated cells, respectively, *n* = 3, *p* < 0.05), thus indicating that PKC is involved in the recycling of the channel to the membrane. These results may suggest that the dynamics of the channels under control and after treatment with calphostin C remain similar during the first hour. However, after 2 h of exposure to calphostin C, an increase in the internalized channels was observed. This effect could be due to (a) an increase in the internalization of the channels after inhibition of PKC, or (b) a decrease in the recycling of the channels to the membrane. As after 1 h of PKC inhibition the behavior of the channels was similar under both experimental conditions, it should be likely that the effect observed after 2 h was due to a malfunctioning recycling of the channels. However, in order to test which hypothesis is correct, immunocytochemical and molecular dynamics in vivo using live cell imaging experiments were performed.

**Figure 4:**
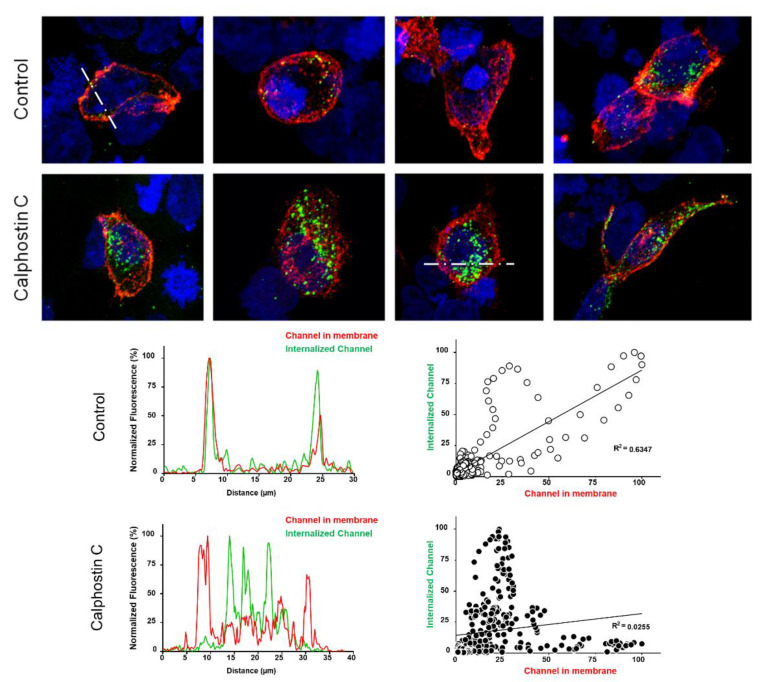
The inhibition of PKC enhances the internalization of K_V_1.5–K_V_β1.3 channels. Representative confocal images of HEK293 cells transfected with K_V_1.5–K_V_β1.3 under control conditions and after treatment with calphostin C. Fluorescence profiles and fluorescence correlation of internalized channels and plasma membrane ones (bottom) in control and after calphostin-C treatment. Note that control cells show mostly a high red signal (plasma membrane channels), whereas those treated with calphostin C present a weaker red signal, but a much greater green signal (internalized channels, which did not recycle to the cell membrane).

To test this hypothesis, immunofluorescence experiments capable of discriminating the internalized channels in control and after calphostin C treatment were performed. To that end, a functionally similar K_V_1.5–HA–K_V_β1.3 construction was used. Figure 4 shows representative confocal images, fluorescence profiles and fluorescence correlation of cells transfected with K_V_1.5–HA–K_V_β1.3 under control conditions and after PKC inhibition with calphostin C. Under control conditions, most cells exhibited cell membrane staining of the K_V_1.5–HA–K_V_β1.3 channels (red color), whereas after PKC inhibition, all stained cells exhibited most of the channels internalized (green color) and less at the cell membrane. In the bottom part of Figure 4, typical profiles of control and calphostin-C-treated cells are shown (left panels). In addition, we show the relationship between the cytoplasm channels and membrane channels under control as well as after treatment with calphostin C.

### 2.3. Inhibition of PKC Disrupts K_V_1.5–K_V_β1.3 Channel Dynamics

**Figure 5:**
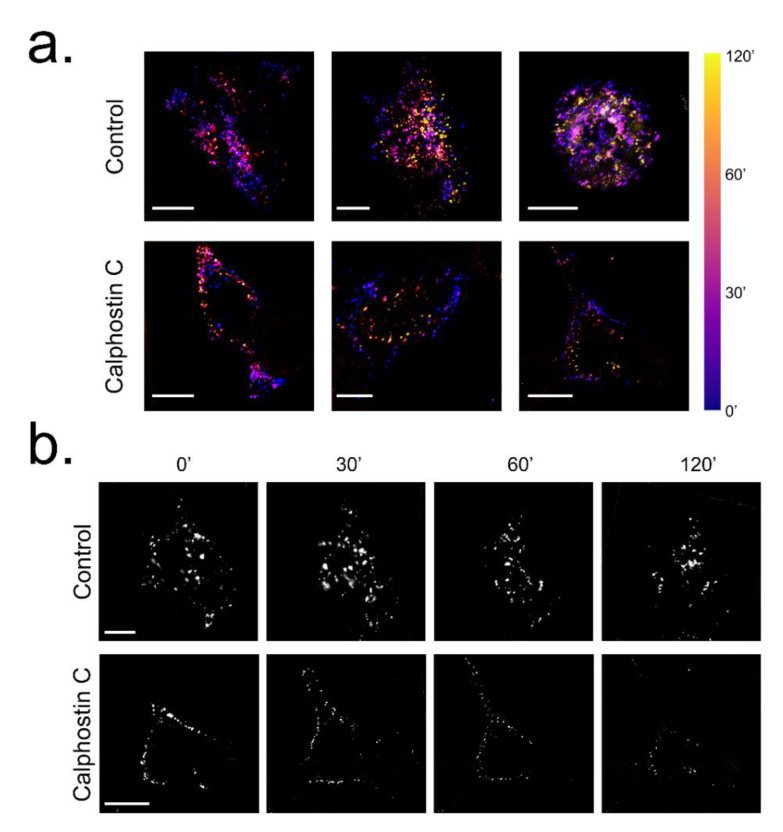
Influence of PKC inhibition on K_V_1.5–HA–K_V_β1.3 mobility in HEK293. (**a**) K_V_1.5–HA labelled by fluorescence staining of live cell under control conditions and after PKC inhibition by calphostin C treatment and visualized at different times. Bar colors show the time from the calphostin-induced PKC inhibition as the beginning of the experiment. (**b**) Frames extracted at 0, 30, 60 and 120 min from Supplemental Video 1A for control and Supplemental Video 2B for calphostin C. Scale bar represents 10 μm in all images.

To analyze the possibility that the PKC inhibition may modify the dynamics of K_V_1.5–HA–K_V_β1.3 channels, live cell imaging assays were performed. Figure 5 shows a summary of the results shown in Supplemental Videos 1A–C (control conditions) and Supplemental Videos 2A–C (calphostin-C-mediated PKC inhibition), where a noteworthy decrease or abolishment, in some cells, of the K_V_1.5–HA–K_V_β1.3 channels dynamics after calphostin-C-mediated PKC inhibition can be observed.

## 3. Discussion

The location of ion channels at precise cell surface areas is a key factor for their proper function. They have active dynamics going back and forth across the cytoplasm, from where they can: a) recycle to the membrane or b) become degraded. The regulation of this traffic provides cells the capacity to accommodate the density of a given transmembrane protein at the plasma membrane. In this work, we have studied the mechanisms by which a decrease in the K_V_1.5–K_V_β1.3 current amplitude is observed after inhibition of PKC. In this article, we demonstrate that the inhibition of PKC produces not only an abolishment of the K_V_β1.3-induced fast inactivation, but also a reduction in the amount of channel protein in the cell membrane, along with a dramatic accumulation of channels into the cytoplasmic compartment. In addition, we show that this accumulation is not due to an increase in the internalization of K_V_1.5–K_V_β1.3 channels, but the result of a reduction in the recycling rate.

The current amplitude generated by K_V_1.5–K_V_β1.3 channels expressed in cells whose PKC was inhibited (either by calphostin C, bisindolylmaleimide II or hispidin) did not exhibit the K_V_β1.3-induced fast inactivation [7,10]. Similar results were found in those experiments in which an antibody anti-RACK1 was present in the internal solution of the patch pipette. All these results suggest that the translocation of activated PKC to the membrane and its binding to RACK1 is a crucial factor for the proper K_V_1.5–K_V_β1.3 channel gating. In addition, when endogenous PKC of the cells was inhibited with calphostin C, a smaller amplitude of the current was observed. This effect can be explained by: (i) a calphostin C-induced degradation of K_V_1.5–K_V_β1.3 channels, or (ii) a decrease in the amount of K_V_1.5–K_V_β1.3 channels in the plasma membrane. The first possibility can be ruled out, taken into consideration the data from the proteinase K experiments, which show that the amount of K_V_1.5–K_V_β1.3 channels present in the cell membrane decrease around 30%. These data are different to those reported with K_V_1.5 channels alone [17], in which PKC activation induced an increase in the internalization of the channels. Taking these data together, we may suggest that the PKC activity is not only involved in the K_V_1.5 channel recycling, but also in the interaction between K_V_1.5 and K_V_β1.3 subunits and the recycling of the resulting channels.

The reduction observed in the amount of channel protein in the plasma membrane after inhibit PKC with calphostin C can be due to either: (i) an increase in the internalization rate of the channels or (ii) a reduction in the channel recycling to the membrane. The biotinylation assays suggest that the internalization of the K_V_1.5–K_V_β1.3 channels mainly occurs during the first hour of the calphostin C treatment, in a similar way to the control conditions. However, at longer times, the number of internalized channels significantly increased. Indeed, fluorescence imaging of live cell experiments showed a dramatic decrease in the recycling to the membrane of the K_V_1.5–K_V_β1.3 channels after PKC inhibition. In these cells, the channels were endocytosed, although their recycling process to the cell membrane was prevented. Moreover, the immunocytochemical experiments show that, whereas most cells under control conditions exhibited K_V_1.5–K_V_β1.3 channels mainly in the cell membrane, those cells treated with calphostin C displayed most of the K_V_1.5–K_V_β1.3 channels in the cytoplasm and, to a lesser extent, at the cell membrane. Rab-GTPases, together with their effector proteins, synchronize intracellular membranous trafficking, including endocytosis, a process through which cells internalize extracellular material, ligands, and plasma membrane proteins and lipids. Rab4 participates in endocytosis through recycling early endosomes, mediating fast recycling process. On the other hand, Rab11 is associated with the recycling endosome and it is involved in the slow recycling of proteins to the plasma membrane [18]. Since PKC inhibition substantially decreases the K_V_1.5–K_V_β1.3 recycling after more than 60 min, we hypothesized that proper K_V_1.5–K_V_β1.3 channels’ recycling is Rab11-mediated. Therefore, all these results suggest that the decrease in the amplitude of the current recorded in cells with PKC inhibition is mainly due to a failing in the normal traffic of K_V_1.5–K_V_β1.3 channels that leads to a decrease in the recycling of these channels to the plasma membrane.

Another explanation for the effects of PKC inhibition in cells expressing K_V_1.5–K_V_β1.3 channels on the traffic to the membrane should be the following. PKC activity can be directly regulated, but it can also be controlled by the activity of other scaffold proteins or its receptor, RACK1. Moreover, Rab-GTPases may also be modulated by other interacting proteins or FIPs. FIPs represent a conserved family of Rab11 effectors known to bridge from Rab GTPases to different molecular motors, ensuring vesicle motility. Indeed, FIP2 interacts with the actin-based myosin Vb motor protein. This interaction is crucial for numerous aspects of FIP2 function, as well as to the regulation of plasma membrane proteins recycling [19,20]. An important issue to be highlighted is that the activities of FIPs are modulated by phosphorylation and phospholipids [19].

There is increasing evidence that demonstrates that classical PKCs regulate trafficking through endocytic and exocytic routes. In fact, these kinases not only regulate ion channels [21,22,23,24,25,26,27] but also the expression of epidermal growth factor receptor (EGFR) and transferrin [28,29,30]. Similarly, serotonin receptor stimulation that activates classical PKCβII induces the retention of serotonin receptors at the endocytic recycling compartment (ERC) [28,31]. Other studies reveal that PMA-induced PKC activation redirects internalized EGFR and protease-activated receptor 1 (PAR-1) from a degradative to a recycling pathway [32]. All these results can stand the molecular basis of the K_V_1.5 channels trafficking, together with previous reports [18,33,34,35,36,37,38,39]. Importantly, quinidine effects on K_V_1.5 channels have been partially attributed to its ability to internalize K_V_1.5 [40]. In addition, we demonstrate that PKC inhibition results in a reduction in K_V_1.5–K_V_β1.3 channels in the cell membrane due to a decrease in the recycle rate of the channel. Thus, the levels and/or the PKC activity may be crucial factors in the antiarrhythmic drug treatment in clinical practice [41,42].

In conclusion, we have characterized the effects of the PKC inhibition on the traffic of the K_V_1.5–K_V_β1.3 channels. This study opens up a variety of possible mechanisms that can help to understand the underlying mechanisms of certain cardiovascular diseases, mostly those involving changes in the PKC expression and/or function.

## 4. Materials and Methods

### 4.1. Plasmids, Cell Culture and Transfection

cDNA codifying for human K_V_1.5 channels and for the K_V_β1.3 subunit was cloned in a pBK-CMV bicistronic plasmid as described [4,5,7]. Briefly, the human K_V_1.5 channel and the K_V_β1.3 subunit were inserted into a pBK-CMV vector, preceded by an IRES sequence, generating a bicistronic messenger RNA [5]. The K_V_1.5–HA construct was kindly provided by Dr. D.J. Snyders (Univ. of Antwerpen, Belgium). HA epitope was digested with *BlpI* and *AflII* and this cDNA digestion product was swapped, thus generating the construction K_V_β1.3–IRES–K_V_1.5–HA. Another construction tagged with a FLAG sequence into the S1–S2 external loop of the K_V_1.5 (between residues 307 and 308) of the K_V_β1.3–IRES–K_V_1.5 cDNA was obtained by the overlapping PCR technique (Table 1) previously described [43].

HEK293 cells were cultured in Dulbecco’s modified Eagle’s medium (DMEM) supplemented with 10% fetal bovine serum, 10 U/mL of penicillin-streptomycin, and 1% nonessential amino acids (all from Sigma-Aldrich, St. Louis, MO, USA). The cells used in this work, passages 2–15 and karyotyping consistent with HEK293 cells, were regularly tested for Mycoplasma contamination. For electrophysiological experiments, cells were cultured in 35 mm plates. Afterwards, cells were transfected with K_V_1.5–K_V_β1.3 channels (0.8 μg) and a reporter plasmid CD8 (1.8 μg). Before experimental use, polystyrene microbeads, pre-coated with an anti-CD8 antibody (Dynabeads M450, Thermo Fisher Scientific, Waltham, MA, USA), were added to the culture plates for 10 min as previously described [6,7,8]. For experiments of immunocytochemistry, 3 μg of other constructions was used (pBK–CMV–K_V_β1.3–IRES–K_V_1.5–HA and pBK–CMV–K_V_β1.3–IRES–K_V_1.5–FLAG). For experiments of protein expression, coimmunoprecipitation and biotinylation, cells were transfected with 8 μg of total amount of cDNA per 100 mm culture dish, of the construction that expresses K_V_β1.3–IRES–K_V_1.5, K_V_β1.3–IRES–K_V_1.5–HA or K_V_β1.3–IRES–K_V_1.5–FLAG proteins. In all cases, transient transfections were performed in HEK293 cells using the FuGENE^®^6-transfection method (Promega, Madison, WI, USA) and the experiments were conducted 48 h post-transfection. In the internalization assays, transfection in both experimental groups was always performed at the same time and with the same transfection cocktail in order to eliminate the experimental variability in the ion channel expression between them. The ratio μg cDNA:μL FuGENE^®^6 was always 1:3.

### 4.2. Internalization Assays by Biotinylation

For these experiments, HEK293 cells were transfected with the pBK–Kvβ1.3–IRES–Kv1.5–FLAG construction 48 h before biotinylation experiments, which was performed by using the Pierce^®^ Cell Surface Protein Isolation Kit (Thermo Fisher Scientific, Waltham, MA, USA), following the manufacturer instructions. Cell surface proteins were biotin-labeled using Sulfo-NHS-SS-biotin and incubated at 4 °C in gentle mixing during 30 min. The unreacted biotin was shielded, and cell culture was warmed at 37 °C to allow channel dynamics that was analyzed at different times. The residual cell surface biotin was removed with MesNa (100 mM sodium 2-mercaptoethanesulfonate, 50 mM Tris, 100 mM NaCl, 1 mM EDTA, 0.2% BSA). Afterwards, cells were washed in cold PBS and lysed with ice-cold lysis buffer. The protected biotin-tagged (endocytosed) channels were pulldown with 25 μL of NeutrAvidin-agarose added to 500 μg of total protein. The amount of protein was resolved by SDS-PAGE (10% polyacrylamide gel) and transferred to PVDF membranes. Internalized K_V_1.5 channels were detected using anti-K_V_1.5 (APC-004, Alomone, Jerusalem, Israel).

### 4.3. Internalization Assays by Immunocytochemistry

Cells were grown in sterilized 12 mm diameter coverslips (Thermo Fisher Scientific, Waltham, MA, USA) and transfected. At 48 h post-transfection, cells were incubated in DMEM (without serum and antibiotics) with anti-HA antibody (1:250) (#NB600-366, Novus Biologicals, Centennial, CO, USA) during 30 min at 4 °C with gentle mixing. Subsequently, internalization of the HA-tagged channels bound to the antibody was facilitated by incubating the cells at 37 °C for 2 h. Afterwards, cells were fixed with 4% paraformaldehyde for 20 min, blocked with goat serum 5% in PBS for 1 h, and incubated with the secondary antibody (goat anti-rabbit Alexa-568, 1:500, Molecular Probes, Invitrogen Corporation, Carlsbad, CA, USA). Then, cells were washed and permeabilized with 0.5% *v*/*v* Triton X-100 and goat serum 5% in PBS for 30 min at 37 °C. Finally, cells were blocked with goat serum 5% in PBS for 1 h and then, the second secondary antibody (goat anti-rabbit Alexa-488, 1:500, Molecular Probes, Invitrogen Corporation, Carlsbad, CA, USA) was added in order to stain the internalized channels.

### 4.4. Proteinase K Digestion Assay: Analysis of Cell Surface Protein

Plasma membrane proteins were digested with proteinase K, as described [14]. Each 35 mm cell plate was incubated with a solution containing (in mM): HEPES-Na 10, NaCl 150, CaCl_2_ 2 (pH 7.4) with or without 200 µg/mL proteinase K (Sigma-Aldrich, St. Louis, MO, USA) at 37 °C for 30 min. Then, cells were harvested and centrifuged at 1000× *g* in a microcentrifuge (4 °C). Digestion with proteinase K was stopped with chilled PBS containing 25 mM EGTA supplemented with Complete Protease Inhibitor Cocktail Tablets (Roche Diagnosis, Manheim, Germany). Cells were washed three times with PBS at 4 °C. Lysates were analyzed by immunoblotting, as described above.

### 4.5. Electrophysiological Recordings and Data Acquisition

The intracellular pipette solution contained the following (in mM): K-aspartate 80, KCl 42, phosphocreatine 3, KH_2_PO_4_ 10, MgATP 3, HEPES-K 5, and EGTA-K 5 (adjusted to pH 7.25 with KOH). The bath solution contained the following (in mM): NaCl 140, KCl 4, CaCl_2_ 1.8, MgCl_2_ 1, HEPES-Na 10, and glucose 10 (adjusted to pH 7.40 with NaOH). Currents were recorded using the whole-cell configuration of the patch-clamp technique by using an Axopatch 200B amplifier and a Digidata 1440A using the pClamp 10 (Molecular Devices LLC., San Jose, CA, USA) and data were stored on a personal computer (Hewlett Packard). Currents were recorded at room temperature (21–23 °C) at a stimulation frequency of 0.1 Hz and low pass-filtered and sampled at 2 kHz. Pipettes were pulled from Narishige (GD1; Narishige Co Ltd., Tokyo, Japan) borosilicate capillary tubes using a programmable patch micropipette puller (P-87; Sutter Instrument Co., Novato, CA, USA) and were heat polished with a microforge (MF-83; Narishige Co Ltd., Tokyo, Japan). The average pipette resistance ranged from 1 to 3 MΩ (*n* = 15). After seal formation, cells were lifted from the bath, and the membrane patch was ruptured with an additional suction. Microcal Origin 2018 (OriginLab Co, Northampton, MA, USA) and the clampfit utility of the pClamp 10 software were used to perform least-squares fitting and data presentation. Voltage dependence of the activation curves was fitted using a Boltzmann equation: *y* = 1/[1 + exp(−(*V* − *V_h_*)/*s*)], where *s* represents the slope, *V* the membrane potential, and *V_h_* the voltage at which 50% of the channels are open.

### 4.6. Live Cell Imaging

Cells were cultured in 35 mm μ-dishes suitable for live cell imaging (Ibidi GmbH, Gräfelfing, Germany), transfected with Kv1.5–HA–Kvβ1.3 construction and labelled as described [34]. Cells were incubated with mouse-anti-HA (1:250, Novus Biologicals, Centennial, CO, USA) at 4 °C for 30 min in DMEM (without serum or antibiotics). Afterwards, cells were washed with cold medium and labelled with goat anti-mouse Alexa-488 (1:500, Molecular Probes, Invitrogen Corporation, Carlsbad, CA, USA) at 4 °C for 30 min. Then, cells were washed with cold medium three times. After that, calphostin C or vehicle was added to perform a time course of PKC inhibition effect on the ion channel dynamics. A Microscope Cell Observer Z1 system (Carl Zeiss MicroImaging GmbH, Oberkochen, Germany) equipped with a controlled environment chamber (37 °C constant temperature, 5% CO_2_ atmosphere) and Camera Cascade 1k was used for live imaging. Images were collected every 5 min using a 63 × Plan Apochromat objective (0–3 h). Finally, time-lapse movies and images were formatted using ImageJ software.

### 4.7. Drugs

Calphostin C (Calbiochem, Merck KGaA, Darmstadt, Germany) was dissolved in ethanol to produce a 100 μM stock solution. Calphostin C was added to achieve a final concentration of 3 μM for 2 h before starting experiments. Hispidin from Calbiochem (Merck KGaA, Germany) was dissolved in DMSO to get a 10 mM stock solution. Hispidin was added to the culture cells at a concentration of 5 μM for 30 min. Anti-RACK1 (sc-17754, Santa Cruz Biotechnology, Dallas, TX, USA) was applied in the internal solution of the micropipette at a concentration of 0.4 µg/µL.

### 4.8. Statistical Analysis

Data are presented as the mean ± SEM (standard error of the mean). Statistical analysis was performed in Microcal Origin 2018 (OriginLab Co, Northampton, MA, USA). Outliers were detected using the Grubb’s test and were excluded from the analysis. Student’s *t*-test or one-way analysis of variance was used to compare two or more than two groups, respectively. Statistical significance was set at *p <* 0.05.

## Figures and Tables

**Table 1:** List of primers used for pBK–CMV–K_V_β1.3–IRES–K_V_1.5–FLAG construction.

N-term	
Forward	5′cggctgggctcagcgatgggcccaaggagccggc 3′
Reverse	5′*ccc***cttgtcatcgtcgtccttgtagtc***ccc*gccagaggcgggggccatgaccccgctgccg 3′
C-term	
Forward	5′*ggg***gactacaaggacgacgatgacaag***ggg*cctacggtggcaccgctcctgcccCGTACG 3′
Reverse	5′ gctcttccttaaggactgccggctcctcgtgatcc 3′

Underlined nucleotides indicate the recognition sequences of restriction endonucleases *BlpI* and *AflII*, respectively. Bold sequences indicate nucleotides corresponding to the FLAG sequence; glycines represented by italics were added to provide flexibility at the front and at the back of the insert; nucleotides in uppercase correspond with the insertion of a punctual and silent mutation, which result in a single *BsiWI* restriction site used as reporter of the insertion.

## Data Availability

The data that support the findings of this study are available from the corresponding author upon reasonable request.

## Supplementary Materials

The following are available online at https://www.mdpi.com/1422-0067/22/3/1336/s1.

## Abbreviations

PKC	Protein kinase C
RACK1	Receptor of activated protein C kinase 1
HA	Hemagglutinin
PMA	Phorbol 12-myristate 13-acetate
